# Evaluation of the Community-Based Chronic Disease Prevention Program *Meta Salud* in Northern Mexico, 2011–2012

**DOI:** 10.5888/pcd11.140218

**Published:** 2014-09-11

**Authors:** Catalina A. Denman, Cecilia Rosales, Elsa Cornejo, Melanie L. Bell, Diana Munguía, Tanyha Zepeda, Scott Carvajal, Jill Guernsey de Zapien

**Affiliations:** Author Affiliations: Cecilia Rosales, Melanie L. Bell, Tanyha Zepeda, Scott Carvajal, Jill Guernsey de Zapien, Mel and Enid Zuckerman College of Public Health, University of Arizona, Tucson, Arizona; Elsa Cornejo, Diana Munguía, El Colegio de Sonora, Hermosillo, Sonora, Mexico.

## Abstract

**Introduction:**

*Meta Salud* is a community health worker–facilitated intervention in Hermosillo, Sonora, Mexico, and was adapted from *Pasos Adelante*, a similar evidence-based intervention developed for a Latino population in the United States–Mexico border region. The objective of this study was to examine outcomes for *Meta Salud* and compare them with outcomes for *Pasos Adelante*.

**Methods:**

This pretest–posttest study took place during 13 weeks among low-income residents of an urban area. The program provided information on topics such as heart health, physical activity, nutrition, diabetes, healthy weight, community health, and emotional well-being; included individual and group activities aimed at motivating behavior change; and encouraged participants to engage in brisk physical activity.

**Results:**

We found significant decreases from baseline to conclusion in body mass index, waist circumference, hip circumference, weight, triglycerides, and low-density lipoprotein (LDL) cholesterol. From baseline to 3-month follow-up, we found significant decreases in body mass index, waist circumference, weight, LDL cholesterol, and glucose, and an increase in high-density lipoprotein cholesterol. Outcomes for *Meta Salud* were similar to those found for *Pasos Adelante*.

**Conclusion:**

The physiological improvements found among participants in *Meta Salud* and comparable changes among participants in *Pasos Adelante* suggest a scalable and effective behavioral intervention for regions of the United States and Mexico that share a common boundary or have similar cultural and linguistic characteristics.

## Introduction

The proportion of total deaths from noncommunicable diseases (NCDs) in Mexico tripled from 23% in 1955 to 75% in 2005 (1). Because of high death rates from cardiovascular disease (2), diabetes (3), and other NCDs, Mexico faces challenges in maintaining a healthy society and a sustainable health care system. These diseases often have similar risk factors, such as lack of physical activity (4), smoking (5), and overweight and obesity (6). Diabetes is a leading cause of death for men and women, accounting for 14% of adult mortality in 2009 (7). According to Mexico’s most recent National Survey on Health and Nutrition, the prevalence of hypertension has increased to 31.6%, and increased almost 20% from 2000 to 2006 (8). Recent health data in Mexico illustrate other challenges to improving public health. Obesity rates in Mexico are some of the fastest growing in the world: 71.3% of adults are obese or overweight (9). Among adults aged 20 to 69, rates of physical inactivity increased by 47.3% from 2006 to 2012, and 58.6% of children and adolescents aged 10 to 14 reported no participation in any organized physical activity in the previous 12 months (10). Regional differences between northern, central, and southern Mexico have implications for health prevention and promotion activities. Although southern Mexico has high rates of poverty (11), maternal and infant mortality, and infectious disease, 4 of the northern border states are among the 5 states with the highest prevalence rates for hypertension (2). The northern region has the highest rates of obesity among the adult population, where average body mass index (BMI) is 28.8, compared with the central region (BMI, 28.0) and southern region (BMI, 28.2) (6).

Mexico recently introduced a Health Promotion Operational Model (12) and Healthy Communities Program (13), aimed at addressing public health challenges from a socioecological perspective. These new models recognize that to decrease mortality, improve quality of life, and address the economic costs of NCDs, public health efforts should emphasize not only the management of NCDs but also a range of strategies that focus on health-promoting behaviors and risk reduction and consider the individual, family, community, social, and public policy levels. This strategy entails the widespread implementation of primary prevention and health promotion programs that are facilitated by community health workers (CHWs) and that engage the population in building the foundation of a healthy life before disease develops.

Prevention and health promotion programs led by CHWs effectively and efficiently decrease risk factors for NCDs among the Hispanic population in the United States and particularly among people of Mexican origin (14), but it is not clear whether these programs apply to other socio-environmental contexts. To evaluate whether the outcomes of these programs could be replicated in northern Mexico, in 2011 and 2012 the Center for Health Promotion in Northern Mexico implemented *Meta Salud*, a community-based NCD primary prevention program facilitated by CHWs working for the State Health Ministry in Hermosillo, Sonora, Mexico.

The Center for Health Promotion in Northern Mexico, a collaborative project between El Colegio de Sonora and the University of Arizona Mel and Enid Zuckerman College of Public Health, is housed at El Colegio de Sonora and is part of the global network of Collaborating Centers of Excellence supported by the US National Heart, Lung, and Blood Institute (NHLBI) and the UnitedHealth Chronic Disease Initiative. These Centers of Excellence, located in 11 developing countries around the world, conduct research to monitor, prevent, or control chronic diseases (www.nhlbi.nih.gov/about/globalhealth/centers/).

The priority of the Center for Health Promotion in Northern Mexico is to develop primary prevention and health promotion programs facilitated by CHWs in community settings to decrease risk factors for NCDs. In 2011, we conducted a scoping review of academic articles that describe community-based programs for the primary prevention of NCDs at the United States–Mexico border and identified several evidence-based programs implemented successfully in the United States, including the University of Arizona’s *Pasos Adelante* program (15), adapted from the NHLBI’s *Su Corazón, Su Vida* program (16) and tested in the United States–Mexico border region from 2005 to 2008. Our review also found a dearth of evidence-based primary prevention models for NCDs in northern Mexico (17). Because of the limited availability of evidenced-based models for Spanish-speaking populations in the region and the extensive experience of the University of Arizona with *Pasos Adelante*, we selected, adapted, and tested this program as *Meta Salud* in northern Mexico, also taking into account that *Pasos Adelante* is an evidence-based intervention that addresses the Centers for Disease Control and Prevention’s “winnable battles” of nutrition, physical activity, and obesity (www.cdc.gov/prc/prevention-strategies/chronic-disease-risks.htm).

The objective of this study was to examine the outcomes of the *Meta Salud* intervention, focusing on changes in anthropometric measurements and clinical biomarkers, and compare these outcomes with those of *Pasos Adelante* (18).

## Methods

This was a nonrandomized, quasi-experimental pretest–posttest study of physiological changes among low-income participants in an urban area in northern Mexico. The *Meta Salud* program consists of 13 weekly educational sessions that provide information about topics such as heart health, physical activity, diabetes, fat and cholesterol, sodium, glucose and sugar, maintaining a healthy weight, building a healthy community, preparing healthy foods, eating healthfully on a budget, and emotional well-being. Each 2-hour educational session consists of group activities and team exercises that motivate participants to change through participative methods that promote the adoption of healthy lifestyle habits. The intervention also includes a group physical activity session 1 to 3 times per week. *Meta Salud* program materials (including the handbook for program implementation and a participant workbook) and instructional videos are available at http://sitios.colson.edu.mx/metasalud.

*Meta Salud* is grounded in social cognitive theory, which promotes self-efficacy and skills and reduces impediments to making healthy behavior changes (19). The adaptations for northern Mexico of the *Pasos Adelante* and *Su Corazón, Su Vida* programs included content modifications that account for sociocultural and institutional differences between Mexico and the United States, as well as structural and methodological modifications aimed at promoting agency and empowerment among participants, such as weekly goals and other strategies, based on the transtheoretical stages-of-change model (20), that promote motivation for change. To make the materials easier for CHWs to use and to reduce the need for extensive training of CHWs, we also made changes to the session format and graphic design of the handbook. All changes were reviewed by experts in chronic disease, nutrition, and public health, as well as community members and CHWs.

### Study setting

The intervention was implemented in 4 low-income neighborhoods in Hermosillo, Sonora, Mexico, during 2011 and 2012, in areas surrounding community health centers (*Centros de Salud*) administered by the Sonora State Health Ministry. The Mexican state of Sonora borders the US state of Arizona. Hermosillo, the capital of Sonora, is a medium-sized city with 784,342 inhabitants (21). According to Mexico’s most recent National Survey on Health and Nutrition, 18.1% of the Sonora population older than 20 years have hypertension, 7.7% have diabetes, and 70.6% of men and 76.9% of women are overweight or obese (22).

### Recruitment

Mexico’s health system includes full-time *promotoras de salud*, CHWs who work in community health centers administered by the Health Ministry. *Meta Salud* was facilitated by 9 CHWs, who conducted their regular duties in the health centers in addition to participating in the research project. They were trained by El Colegio de Sonora and University of Arizona research staff to facilitate the *Meta Salud* intervention and received an additional stipend for their participation in the research project. The CHWs worked in 2-person teams to facilitate 4 simultaneous groups during 2 intervention cycles. Training was provided before each intervention cycle.

The CHWs recruited 15 to 20 participants for each intervention group from the neighborhoods surrounding their health centers. Sessions were held in various spaces, including schools, private homes, a police station, and the health centers. Recruitment strategies included contacting community groups (eg, parent groups at the local school) and going door-to-door.

### Study design

The study examined the outcomes of anthropometric measurements, laboratory tests, and a lifestyle questionnaire. Measures were taken, with prior informed consent from all participants, at baseline (during the first or second week of the intervention), at the conclusion of the intervention, and 3 months after the intervention ended (3-month follow-up). Evaluation and intervention protocols were approved by the ethics committee at El Colegio de Sonora.

### Measures

To compare *Meta Salud* outcomes with those of the *Pasos Adelante* intervention in Douglas, Arizona (n = 217), we used the same questionnaire and measurement protocols. The questionnaire covered physical activity, eating habits, physical and mental health, and sociodemographic information. (The findings on physical activity, eating habits, and mental health will be published elsewhere.) The anthropometric measurements included waist and hip circumference for calculating waist–hip ratio and height and weight for calculating BMI (kg/m^2^); these were measured in triplicate, and a fourth measurement was taken if the previous 3 differed by more than 1 cm. The clinical biomarkers used for blood analysis were fasting blood glucose, high-density lipoprotein (HDL) cholesterol, low-density lipoprotein (LDL) cholesterol, total cholesterol, and triglycerides. All members of the research team who participated in collecting evaluation data were trained to administer the lifestyle questionnaire and collect anthropometric measurements. A certified laboratory and phlebotomist collected blood samples.

### Statistical methods

#### Primary analysis

We compared the baseline characteristics of those with complete data with the characteristics of those who had any missing data by using *t* tests and χ^2^ tests. Linear mixed models were used to estimate the differences between baseline and both follow-ups for all continuous outcomes, adjusting for age and using participant as a random effect to account for within-participant correlation due to repeated measurements. Time was used as a categorical variable, which reduces the likelihood of model misspecification (23). All available outcome data were used in the mixed models, which are robust in the presence of missing data (24). We calculated outcomes as differences from baseline and 95% confidence intervals (CIs).

We compared *Meta Salud* with *Pasos Adelante* by graphing the standardized mean difference between outcomes at baseline and both follow-ups (calculated as the difference in means divided by the standard deviation of the mean difference).

#### Sensitivity analysis

Multiple imputation was used to explore the robustness of the outcomes to assumptions about missing data. Twenty multiply-imputed complete data sets were created using SAS Proc MI (SAS Institute Inc). The imputation model included age and each outcome at each time point. Each imputed data set was analyzed with mixed models as described above and combined using Proc MIAnalyze (SAS Institute Inc).

## Results

**Figure 1:**
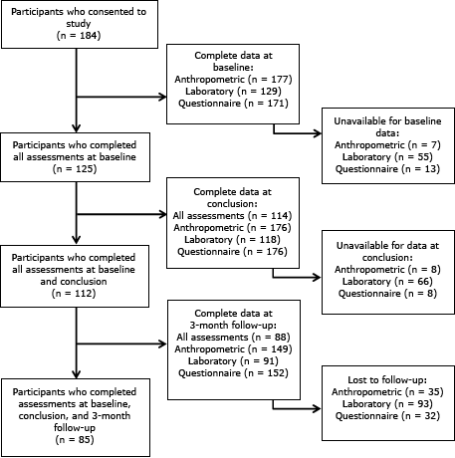
Flow diagram of participants in the evaluation study for *Meta Salud*, a 13-week community-based program for the primary prevention of chronic disease conducted in Hermosillo, Sonora, Mexico, 2011–2012.

Of the 265 participants initially recruited, 184 consented to participate in the study to evaluate the intervention, and 166 (62.5%) successfully completed the program (ie, they attended at least 8 of the 13 weekly educational sessions). However, we did not have all measurements, particularly the laboratory measurements, for all 166 participants: we had complete baseline data for 125; complete baseline and conclusion data for 112; and complete baseline, conclusion, and 3-month follow-up data for 85 (Figure 1).

Of the 171 participants who completed the baseline questionnaire, 98% were women and 85% were married; mean age was 41.5; 90% had less than a high school education; 98% had some type of health coverage; 61% had a family member with diabetes; and 17% had received a diagnosis of diabetes (Table 1). Most (87%) had lived in the community for more than 10 years. We found no significant differences between the 85 participants who had complete data and the 86 who had 1 or more missing assessments.

**Table 1:** Sociodemographic and Health Characteristics of *Meta Salud* Participants and Comparisons of Participants Who Completed All Assessments With Those Who Missed ≥1 Assessment, Hermosillo, Sonora, Mexico, 2011–2012^a^

Characteristic	Baseline Participants (n = 171)^b^	Participants Who Completed All Assessments (n = 85)	Participants Who Missed ≥1 Assessment (n = 86)	*P* Value^b^
**Demographic**				
**Age, mean (SD), y**	41.5 (10.6)	42.7 (10.9)	40.3 (10.3)	.15
**Female sex**	168 (98)	85 (100)	83 (98)	—
**Marital status**				
Married	147 (85)	74 (87)	73 (87)	.68
Single/divorced/widowed	24 (15)	11 (13)	13 (13)	.68
**Education**				
Some elementary	27 (16)	11 (13)	16 (19)	.40
Completed elementary	26 (15)	10 (12)	16 (19)	.40
Some high school	100 (59)	53 (62)	47 (55)	.40
Completed high school	11 (6)	6 (7)	5 (7)	.40
Post high school	7 (4)	5 (6)	2 (6)	.40
**Currently employed**	62 (36)	27 (32)	35 (41)	.22
**Has health insurance**	167 (98)	84 (98)	83 (98)	—c
**Length of residence in community, y**				
<5	8 (5)	4 (5)	4 (5)	.52
6-10	14 (8)	9 (11)	5 (6)	.52
>10	149 (87)	72 (85)	77 (90)	.52
**Born in Mexico**	126 (74)	64 (75)	62 (72)	.63
**Health Status**				
**Have family members with diabetes**	105 (61)	55 (65)	50 (58)	.38
**Given a diagnosis of diabetes**	29 (17)	14 (16)	15 (17)	.87
**Length of time with diabetes, y**				
<1	6 (221)	4 (29)	2 (13)	—c
1–5	7 (24)	3 (21)	4 (27)	—c
6–10	6 (21)	2 (14)	4 (27)	—c
>10	7 (24)	3 (21)	4 (27)	—c
Do not know	3 (10)	2 (14)	1 (7)	—c
**Body mass index (kg/m^2^)**				
>25.0	24 (24)	13 (16)	11 (12)	.07
25.0–29.9	52 (30)	31 (38)	21 (23)	.07
30.0–39.9	81 (47)	31 (38)	50 (54)	.07
>40	17 (10)	6 (7)	11 (12)	.07
**Have heart disease**	12 (7)	7 (8)	5 (6)	.54
**Have high blood pressure**	52 (30)	22 (26)	30 (35)	.20
**Have high cholesterol**	38 (22.2)	20 (24)	18 (21)	.68
**Have asthma**	12 (7)	5 (6)	7 (8)	.56
**Current smoker**	27 (15.8)	10 (12)	17 (20)	.31

Abbreviation: SD, standard deviation.

a All values are number (percentage) unless otherwise indicated.

b Although 184 patients consented to participate, only 171 completed the baseline questionnaire.

c χ^2^ tests not conducted because of small sample size.

We found significant changes from baseline to conclusion in BMI (0.26 [95% CI, 0.10–0.42]), waist circumference (0.91 [95% CI, 0.25–1.57] cm), hip circumference (0.56 [95% CI, 0.02–1.09] cm), weight (0.63 [95% CI, 0.24–1.02] kg), LDL cholesterol (7.93 [95% CI, 1.02–14.8] mg/dL ), and triglycerides (−26.4 [95% CI, −40.4 to −12.4] mg/dL) (Table 2). From baseline to 3-month follow-up, we found significant changes in BMI (0.26 [95% CI, 0.09–0.43]), waist circumference (1.00 [95% CI, 0.30–1.69] cm), waist-to-hip ratio (0.007 [95% CI, 0.001–0.01]), weight (0.66 [95% CI, 0.24–1.11] kg), total cholesterol (14.2 [95% CI, 6.6–21.8] mg/dL), HDL cholesterol (−11.1 [95% CI, −14.1 to −8.1] mg/dL), LDL cholesterol (21.6 [95% CI, 14.0–29.2] mg/dL), and glucose (7.55 [95% CI, 0.08–15.0] mg/dL) (Table 2). The sensitivity analyses using multiple imputation showed similar outcomes in significance and effect sizes.

**Table 2:** Health Outcomes for Participants in *Meta Salud* at Baseline, Conclusion, and 3-Month Follow-up, Hermosillo, Sonora, Mexico, 2011–2012

Outcome	Adjusted^a^ Mean (SD)			Change (95% CI)			
	Baseline (N = 177)	Conclusion (n = 176)	3-Month Follow-up (n = 149)	Baseline Minus Conclusion	*P*	Baseline Minus 3-Month Follow-up	*P*
**Anthropometric**							
Body mass index, kg/m^2^	31.3 (6.2)	31.0 (6.4)	31.0 (6.6)	0.26 (0.10 to 0.42)	.001	0.26 (0.09 to 0.43)	.002
Waist circumference, cm	97.6 (13.4)	96.7 (13.2)	96.6 (13.5)	0.91 (0.25 to 1.57)	.007	1.00 (0.30 to 1.69)	.005
Hip circumference, cm	109.7 (11.8)	109.1 (11.9)	109.5 (12.1)	0.56 (0.02 to 1.09)	.04	0.21 (−0.5 to 0.78)	.46
Waist-to-hip ratio	0.89 (0.07)	0.89 (0.07)	0.88 (0.07)	0.004 (−0.002 to 0.01)	.20	0.007 (0.001 to 0.01)	.02
Weight, kg	77.3 (15.6)	76.7 (15.9)	76.7 (15.8)	0.63 (0.24 to 1.02)	.002	0.66 (0.24 to 1.11)	.002
**Clinical biomarkers, mg/dL^b^**							
Total cholesterol	190.9 (44.5)	185.2 (35.3)	177.7 (32.1)	5.66 (−1.2 to 12.6)	.11	14.2 (6.6 to 21.8)	<.001
HDL cholesterol (mg	51.6 (16.6)	51.7 (16.0)	62.6 (17.0)	−0.15 (−2.9 to 2.6)	.91	−11.1 (−14.1 to −8.1)	<.001
LDL cholesterol	109.5 (45.0)	101.5 (33.6)	87.8 (27.3)	7.93 (1.02 to 14.84)	.02	21.6 (14.0 to 29.2)	<.001
Triglycerides	133.9 (80.3)	160.3 (77.1)	138.8 (67.4)	−26.4 (−40.4 to −12.4)	<.001	−5.0 (−20.3 to 10.4)	0.53
Glucose	114.5 (49.1)	113.3 (45.9)	106.9 (48.6)	1.17 (−5.60 to 7.94)	.73	7.55 (0.08 to 15.0)	.05

Abbreviation: SD, standard deviation; CI, confidence interval; HDL, high-density lipoprotein; LDL, low-density lipoprotein.

a Means derived from the linear mixed model described in the Methods section.

b Sample sizes are n = 129 at baseline, n = 118 at conclusion, and n = 91 at 3-month follow-up.

**Figure 2:**
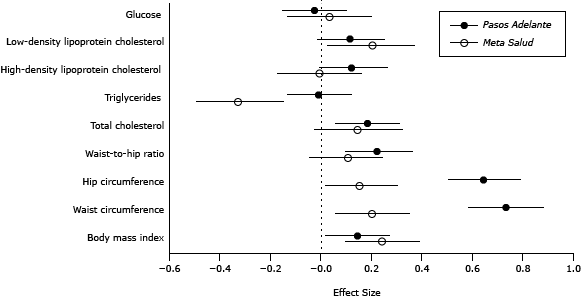
Comparison of outcomes from 2 chronic disease prevention programs: *Meta Salud* (Northern Mexico, 2011–2012) and *Pasos Adelante* (Southern Arizona, 2005–2008). Values are standardized effect sizes of the difference from baseline to conclusion and 95% confidence intervals. **Table:** 

The outcomes for our intervention and those of *Pasos Adelante* were similar, except for changes in triglycerides, which were larger in the *Meta Salud* program, and changes in hip and waist measurements, which were larger in *Pasos Adelante* (Figure 2).

Outcome	Study	Effect Size (95% Confidence Interval)
Body mass index	Pasos Adelante	0.14 (0.01 to 0.27 )
	Meta Salud	0.24 (0.09 to 0.39)
Waist circumference	Pasos Adelante	0.73 (0.58 to 0.88)
	Meta Salud	0.20 (0.05 to 0.35)
Hip circumference	Pasos Adelante	0.64 (0.50 to 0.79)
	Meta Salud	0.15 (0.01 to 0.30)
Waist-to-hip ratio	Pasos Adelante	0.22 (0.09 to 0.36)
	Meta Salud	0.10 (−0.05 to 0.24)
Total cholesterol	Pasos Adelante	0.18 (0.05 to 0.31)
	Meta Salud	0.14 (−0.03 to 0.32)
Triglycerides	Pasos Adelante	−0.01 (−0.14 to 0.12)
	Meta Salud	−0.33 (−0.50 to −0.15)
High-density lipoprotein cholesterol	Pasos Adelante	0.12(−0.01 to 0.26)
	Meta Salud	−0.01 (−0.18 to 0.16)
Low-density lipoprotein cholesterol	Pasos Adelante	0.11(−0.02 to 0.25)
	Meta Salud	0.20 (0.02 to 0.37)
Glucose	Pasos Adelante	−0.03(−0.16 to 0.10)
	Meta Salud	0.03 (−0.14 to 0.20)

## Discussion

Participants who completed the *Meta Salud* program demonstrated important physiological changes from baseline to 3-month follow-up, including a significant decrease in BMI, waist circumference, weight, LDL cholesterol, and glucose; they also had a significant increase in HDL cholesterol. The physiological changes observed in *Meta Salud* and *Pasos Adelante* were similar. Additionally, the changes were similar to those found in a similar intervention among a Hispanic population in the Texas border region (25).

Our study has several limitations. First, it was not a randomized trial and did not have a comparison group. Although analysis and design sought to consider confounders, recruitment may have appealed mostly to people who were already beginning to modify their lifestyles. Second, during the *Meta Salud* intervention, a national media campaign and 2 health-care system interventions (*5 Pasos*; PrevenIMSS; and *Es Tiempo*, *¡Cuídate!*) were being implemented to encourage an active lifestyle and healthful eating habits. Third, this article does not include analysis of data on health status, physical activity, or eating habits. Finally, participation was affected by numerous issues: 37.4% of participants did not complete the program because of, for example, lack of child care, conflicting work schedules, lack of transportation, family emergencies, or an inability to commit to a 13-week program. These issues also reduced the number of participants for whom we had complete measures for program evaluation, particularly blood test results. Although a sensitivity analysis can estimate the potential effect of missing data on outcomes, the challenge remains to overcome the barriers that prevent participants from completing the program. Other challenges are how to increase participation among men, sustain training and improve working conditions for CHWs, and advocate for better urban infrastructure for physical activity, all areas for future research. Despite these limitations and challenges, the overall goal of the study was to determine whether an existing chronic disease prevention and health promotion intervention led by CHWs (*Pasos Adelante*), which resulted in positive changes among a Mexican-origin population in the US border region, can be adapted for and produce similar results among a Mexican population in northern Mexico. Our results show that *Meta Salud* can produce the same kinds of results as those produced by *Pasos Adelante*.

That CHWs are an established part of the Health Ministry’s infrastructure makes *Meta Salud* potentially scalable and sustainable by the Ministry of Health. The challenges to this potential include achieving buy-in at the federal level, leveraging resources to reposition the health care system from an almost exclusive emphasis on disease management and care toward a greater emphasis on primary prevention and health promotion, and training CHWs to facilitate NCD-prevention programs such as *Meta Salud*.

The physiological improvements among participants in *Meta Salud* and comparable improvements in *Pasos Adelante* suggest a scalable and effective behavioral intervention not only for the states that share the boundary between Mexico and the United States but also for other states in both countries and other regions with similar cultural and linguistic characteristics. Our study makes a strong case for collaboration between Mexico and the United States to develop shared solutions that are relevant to the region and beyond, rather than each country focusing only on its own population and suspending efforts at its borders. Our findings also suggest a model program for preventing NCDs, a chief long-term public health problem among Mexicans and among people of Mexican origin in the United States, the largest Hispanic subgroup in that country.
